# Quality of Life Assessment in Multiple Sclerosis: Different Perception between Patients and Neurologists

**DOI:** 10.3389/fneur.2017.00729

**Published:** 2018-01-11

**Authors:** Maria C. Ysrraelit, Marcela P. Fiol, Maria I. Gaitán, Jorge Correale

**Affiliations:** ^1^Institute for Neurological Research Dr. Raúl Carrea, FLENI, Buenos Aires, Argentina

**Keywords:** quality of life, health-related quality of life, patient reported outcome, multiple sclerosis, SF-36

## Abstract

**Background:**

In recent years, neurologists are noticing that evaluation of multiple sclerosis (MS) patients based on combining relapses, disability progression, and magnetic resonance imaging activity may be insufficient to adequately assess suboptimal responses to available therapy. Inclusion of quality of life (QoL) parameters may contribute to breach this gap.

**Objective:**

To evaluate agreement levels between doctor and patient perception of QoL in MS.

**Methods:**

A total of 700 MS patients and 300 neurologists were invited to participate in a cross-sectional study by answering an e-mail questionnaire. The survey collected information on demographical data and included the Short Form questionnaire (SF-36). After completing the questionnaire, patients were given a standard written description of each of the subdomains assessed by SF-36 and asked to identify which three were the most important determinants of their overall health-related QoL.

**Results:**

A total of 135 neurologists and 380 MS patients responded the survey. Study population mean age was 42.1 ± 10.5 years, with 61% presenting relapsing-remitting MS. SF-36 results were physical function 68.4 ± 30, physical role limitation 56.8 ± 41.7, vitality 47.6 ± 21.4, pain 71.2 ± 26.1, social function 72.6 ± 28.6, emotional role limitation 63.2 ± 39.8, mental health 60 ± 14.1, and general health 55.8 ± 22. Doctors considered physical function (75%) and physical role limitation (70%) as the most important QoL determinants in MS, followed by emotional role limitation (52%). Patients however, assigned significantly different levels of importance to physical function (58%), and physical role limitation (46%) and considered vitality (52%) more important than their physicians (*p* < 0.001). Important to note, the results of SF-36 questionnaire were highly correlated with the perception gap between patients and neurologists (*r* = 0.89; *p* = 0.0004).

**Conclusion:**

Concerns on QoL in MS are different for patients and physicians. It is essential to enhance communication in order to better understand actual patient needs.

## Introduction

Multiple sclerosis (MS) is a chronic inflammatory disease of the central nervous system leading to demyelination and neurodegeneration. It is the second cause of disability in young adults after car accidents.

Quality of life (QoL) assessment has many apparent merits in chronic illness outcome measurement. It can be used to measure incremental improvement rather than complete cure, considering a wide range of aspects of daily living, it is centered around the patient and can be used across various medical disciplines (1).

WHO defines QoL as an individual’s perception of life in the context of the culture and values system they live in, as well as in relation to their goals, expectations, standards and concerns. Some definitions of QoL focus on subjective patient perspective of health status (2, 3), whereas other constructs are broader and include objective indicators of health, housing, and other material circumstances (4). Most researchers believe that both subjective and objective information is necessary to establish the construct (5). Subjective and objective appraisals of QoL represent different data but both play a role in assessment (6). Thus, most QoL models reflect a multidimensional conceptual approach, frequently including physical, mental, social, and functional aspects of health. Beyond these core dimensions, many measures incorporate disease- or treatment-specific variables (7). However, different approaches to QoL measurement frequently tend make disease assessment more cumbersome, complicating implementation of a single assessment tool worldwide, across languages, cultures, and individual researcher preferences (8).

Health-related QoL (HRQoL) represents the link between QoL and individual health status. It is generally considered to be multidimensional, encompassing physical and occupational function, emotional status, social interaction, and somatic sensations (3). Thus, HRQoL questionnaires aim to provide a broad, comprehensive and subjective measure of disease impact (including aspects of health that cannot be evaluated using observer-based measures) as well as impact of treatment and presence of side effects.

Multiple sclerosis significantly affects QoL, interfering with a patient’s ability to work, pursue leisurely activities, and execute daily life tasks. Although different studies have investigated QoL in MS patients (9–11), results may vary across regions, cultures and health care systems.

Traditionally, physicians have singled out physical and emotional symptoms as the most important negative aspects of illness, equating health to absence or reduction of disease, and not to complete physical, mental, and social well-being (12). Indeed, previous studies have already shown that patients and doctors disagree on which health domains are most important in MS (10, 11). Combination of relapses, physical disability progression and magnetic resonance imaging (MRI) disease activity reflect only part of the impact that MS has on a patient’s daily life. In recent decades HRQoL measurements are also being considering increasingly relevant for the evaluation of disease progression, treatment response, and level of assistance required by MS patients (8).

In fact, in recent years, researchers are recommending evaluation of HRQoL be included in the definition of No Evidence of Disease Activity (13).

In this study, we analyzed different factors affecting HRQoL in a cohort of Argentine MS patients. Additionally, we assessed differences between patient and doctor perception of HRQoL in MS.

## Materials and Methods

We designed a cross-sectional study implemented through an e-mail questionnaire sent out to 700 patients from the Institute for Neurological Research Dr. Raúl Carrea in Buenos Aires, Argentina and from an Argentine MS patients association (ALCEM), member of the MS International Federation between February and March 2016. The study was prepared following Strengthening the Reporting of Observational studies in Epidemiology (STROBE) statement guidelines for cross-sectional studies (14).

Patients were eligible if they fulfilled 2010 Mc Donald Criteria (15) and had not experienced acute neurological relapses in the 30 days prior to answering the questionnaire.

The survey was specifically designed to study demographical data and medical aspects of disease. Questions included information on: MS type, disease duration, walking ability, and use of disease-modifying therapies.

Finally, patients answered the Short Form questionnaire (SF-36), a HRQoL instrument which has been used extensively to quantify HRQoL changes in MS patients (10, 16, 17) and has also been validated in our region (18). This instrument addresses health concepts relevant to MS patients from the patient’s perspective. There is no single overall score; instead SF-36 generates eight subscales scores and two summary ones. Subscales include: physical functioning, role limitations due to physical problems, body pain, general health perceptions, vitality, social functioning, role-limitations due to emotional problems, and mental health. Summary scores correspond to physical and mental component totals. After completing the questionnaire, patients were given a standard written description of each of the subdomains assessed by SF-36 and asked to identify which three were the most important determinants of their overall HRQoL.

Invitations to participate in the study were sent out to all members of the Argentinean Society of Neurology through their weekly online newsletter. The society has 1,200 active members and viewing rates for the newsletter average 25%. Of the approximately 300 neurologists who read the survey, 135 agreed to participate. Physicians were asked to select which three of all SF-36 domains were the most important determinants of patient HRQoL. Physician demographics were also assessed (gender, years of specialty practice and public vs. private sector activity).

Study protocol was approved by the Institutional Ethics Committee, and written informed consent obtained from all participants before entering the study.

### Statistical Analysis

Descriptive statistics was used to summarize baseline patient demographics and MS clinical status. Data of the three most important SF-36 domains were expressed as the proportion of patients and neurologists who identified a domain as one of the three most important. All statistical analyses included the *Z*-test at a confidence level of 90% with a one-tail test of significance. All analyses were performed using software package SPSS for Windows (version 20).

## Results

A total of 380 individuals with MS and 135 neurologists answered the questionnaire.

Because a recent relapse could transiently modify HRQoL perception, patients experiencing exacerbations during the previous month were excluded from the analysis (*n* = 34).

Clinical and demographical data of patients and clinicians are summarized in Tables 1 and 2, respectively.

**Table 1:** MS patients’ characteristics.

Total patients (*n*)	380
Excluded (*n*)	34
Total included (*n*)	346
Age (years), mean ± SD	42.1 ± 10.5
Female (*n*)	252 (72.8%)
Mean disease duration (years), mean ± SD	8.5 ± 6.5
Type of MS (*n*)	
RRMS	211 (61%)
SPMS	20 (5.8%)
PPMS	11 (3.2%)
Unknown	104 (30%)
Treatments (*n*)	
Interferons	158 (45.7%)
Glatiramer acetate	43 (12.4%)
Natalizumab	24 (6.9%)
Oral drugs (teriflunomide/fingolimod)	83 (24%)
No treatment	38 (11%)
Walking performance	
Normal,	184 (53.2%)
Walk without assistance up to 1,000 m	104 (30%)
Requires unilateral or bilateral assistance	36 (10.4%)
Restricted to wheelchair	22 (6.4%)

*RRMS, relapsing remitting multiple sclerosis; SPMS, secondary progressive multiple sclerosis; PPMS, primary progressive multiple sclerosis*.

**Table 2:** Neurologist’s demographics.

Total clinicians (*n*)	135
Female (%)	52.5
Years of specialty practice	
>15	45%
10–15	17.5%
5–10	20%
<5	17.5%
Main practice	
Private institution	55%
Public institution	37.5%
Private office	7.5%

### SF 36 Results

**Figure 1:**
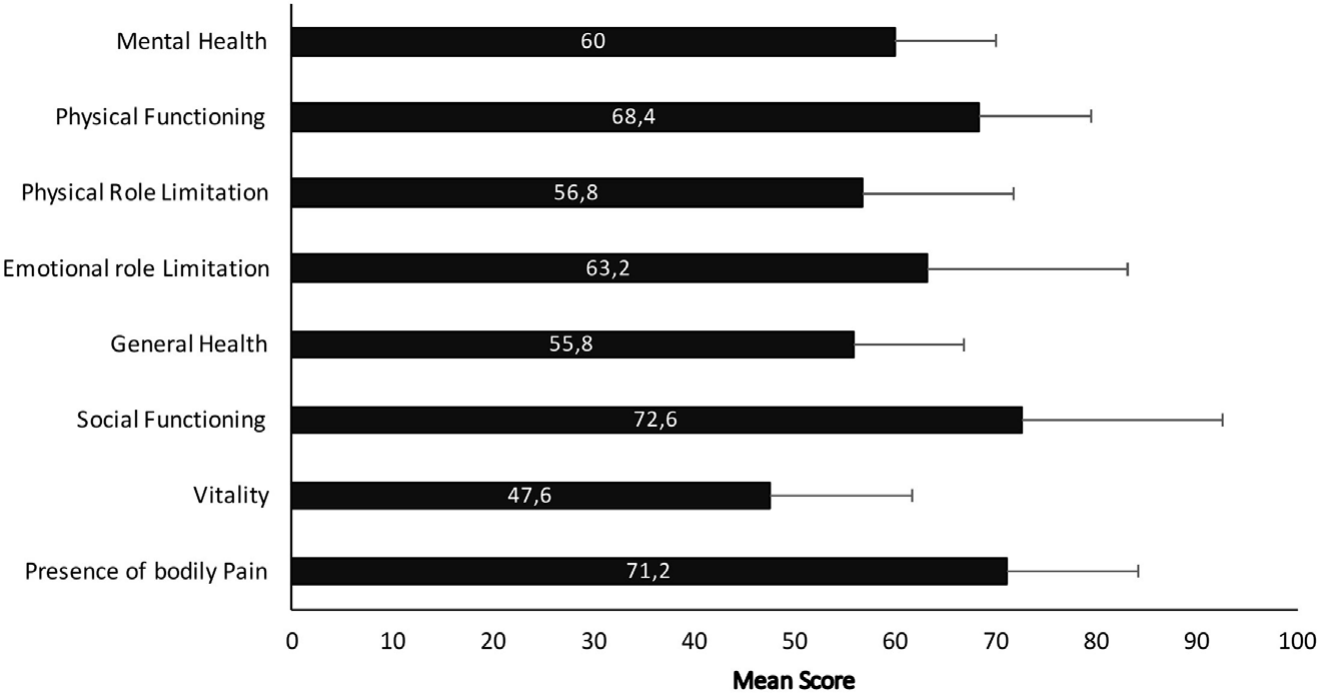
Mean scores of SF-36 subdomains scale in multiple sclerosis patients.

Mean scores of each SF-36 domains are shown in Figure 1. When patients were asked about their general health status, 78.9% (*n* = 273) reported a positive view (good, very good, or excellent), a result associated with younger patient age, recent diagnosis (in the last year), female gender, and better walking performance. Older age was associated with worse outcomes for all SF-36 domains, except mental health (*p* < 0.04). Unrestricted walking was associated with better SF-36 results (*p* < 0.01). No differences were observed in patient walking scores prior to becoming restricted to a wheelchair. Almost 60% of patients reported limitations when carrying out intense activities such as running, lifting heavy weights or practicing high performance sports; and 50% had reduced their daily work activities (ADL). 90% of patients referred fatigue, constant, or during most of the day for 61 and 50% referred exhaustion during the last month.

These results were not related to current disease modifying treatments [interferons (IFNs) vs. natalizumab (NZB) *p* = 0.14; IFN vs. glatiramer acetate (GA) *p* = 0.59; IFN vs. oral therapies (OTs) including teriflunomide or fingolimod *p* = 0.09; NZB vs. GA *p* = 0.1; NZB vs. OT *p* = 0.68; GA vs. OT *p* = 0.11].

### Patient versus Doctor Concerns Regarding HRQoL

Doctors considered physical function (75%) and physical role limitation (70%) as the most important determinants of overall HRQoL in MS, followed by emotional role limitation (52%). We found physician answers were not affected by gender, work-place, or years of specialty experience.

Although patients also considered physical function (58%) and role limitation (46%) to be important aspects of their HRQoL, results were significantly different from those reported by neurologists (*p* < 0.001). Patients also considered vitality (52%), general health (30%) and presence of body pain (30%) important, all aspects not considered as relevant by the majority of neurologists (52 vs. 20%; 30 vs 12%, and 30 vs. 19%; *p* < 0.0001).

**Figure 2:**
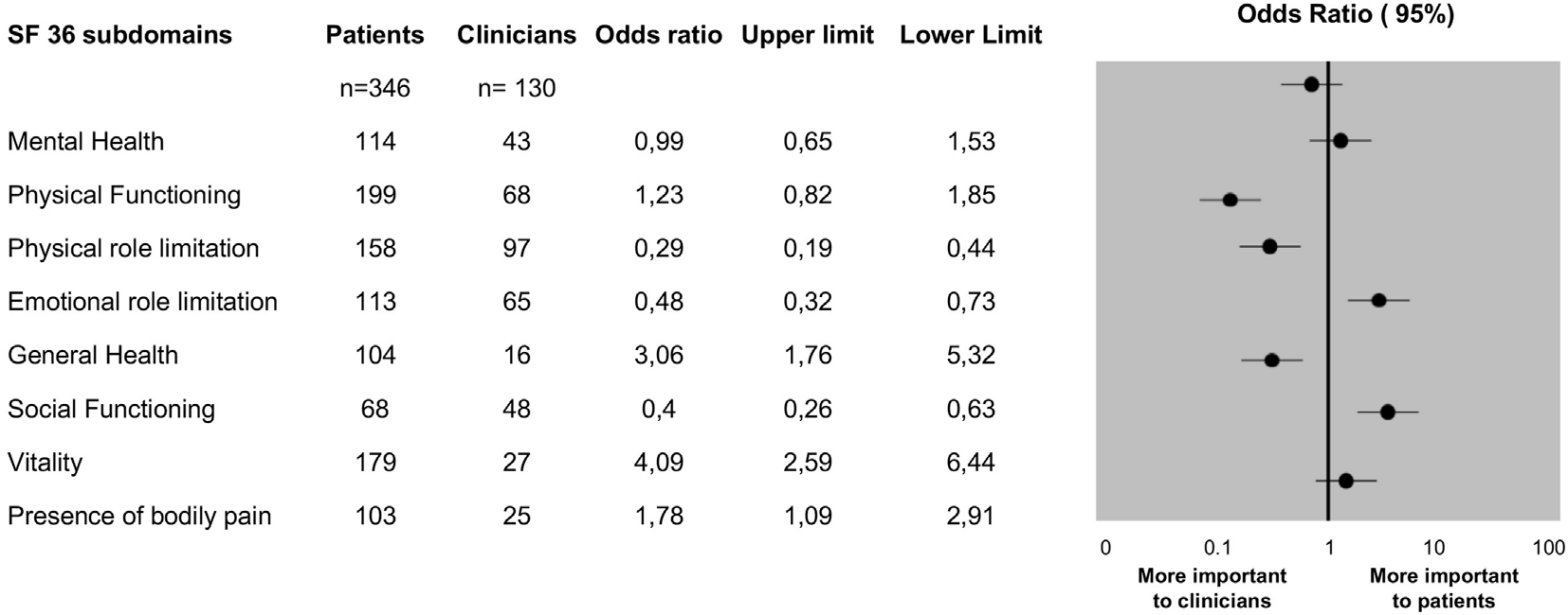
Subdomains of the SF-36 reported to be important by patients and neurologists.

Distribution rates of each of the eight domains of SF-36 questionnaire reported to be important by patients and neurologists are shown in Figure 2.

Important to note, the results of SF-36 questionnaire were highly correlated with the perception gap between patients and neurologists (*r* = 0.89; *p* = 0.0004).

When scores were analyzed according to patient mobility, we found that fully ambulatory patients referred vitality, mental health and physical function (in that order) as the most important aspects influencing HRQoL; whereas patients with minimal disability (requiring assistance up to walk 1,000 m) singled out physical function, physical role limitation followed by vitality as the most relevant subdomains (*p* < 0.001).

## Discussion

The concept of QoL and HRQoL and its determinants has evolved since the 1980s to encompass aspects of overall QoL clearly shown to affect health—either physical or mental. Other factors also predicted to affect HRQoL include: disease symptoms, adverse drug reactions, employment, economic factors, pursuit of leisurely activities, and execution of daily life tasks (19). Although HRQoL data are increasingly used as secondary end points in MS clinical trials, they have been applied much less commonly to clinical care practice. This information would significantly improve patient physician communication regarding approaches to disease management and living with a chronic disease. Arguably, doctors offer treatment to patients for three reasons: (1) to prolong life, (2) prevent morbidity, and (3) to recover overall wellbeing. To assess how well they accomplish the third of these goals clinicians have come to depend increasingly on patient HRQoL assessment (20).

For this reason, researchers are now recommending HRQoL assessments be included together with other parameters, both clinical (relapses, MRI activity, EDSS progression, cognitive function, and fatigue) and biological (atrophy and neurofilament levels) when evaluating suboptimal response to MS treatment (21). In fact, the Institute of Medicine currently considers inclusion of individual patient perspective a key element to improving healthcare care outcome (22).

In this study, we found disagreement between MS patients and treating physicians regarding factors affecting HRQoL. While clinicians focused mainly on physical aspects, patients considered vitality, pain, as well as general, emotional, and mental health also relevant.

Nevertheless, in comparison to previous studies (10, 11) we found some differences. Doctors consider emotional role limitation in MS patients more relevant (50% in our study vs. 8% in a study by Rothwell) and are less concerned about physical decline (52 vs. 80%, respectively). Although study result discrepancies could partially be attributed to social, cultural, or healthcare system differences, they may also represent differences in treating physician outlook, indicating more importance is being assigned to limitations caused by MS, other than physical function.

Furthermore, comparison between the present study and other published series (10) such as the one by Rothwell showed more importance was assigned to vitality (52 vs. 30.9%) and pain (30 vs. 11.9%), and less to mental health (33 vs. 59.5%), respectively. Possible explanations for these differences might include the fact that: (1) patient-reported QoL in MS differs between cultures and countries, (European, and Canadian vs. Latin American) (23); (2) warmer weather conditions in Latin America have been linked to higher MS frequency and more impact of fatigue on HRQoL (24); and (3) the number of patients and doctors surveyed was greater in this study which may have altered statistical results.

As expected, SF 36 results in this study were better in fully ambulatory patients (25). Predictably, patients with mobility limitations of any level, singled out physical performance as the most relevant factor affecting HRQoL. However, perceived HRQoL decline was similar between patients able to walk without assistance up to 1,000 m and those requiring bilateral support, emphasizing the impact that even minimal disability has on daily living for MS patients.

As mentioned, most subjects referred fatigue as a limitation to activities of daily living. Mechanisms underlying fatigue in MS are unknown nor have potential links to current treatments been ruled out (26–28). Because we had observed some treatments seemed to have a negative impact on fatigue in clinical practice, we looked for correspondence between disease-modifying therapies and either fatigue or SF-36 scores. However, no correlation was found.

Of note, classical trials evaluating treatment efficacy increasingly incorporate patient-reported outcomes (PROs) to support labeling claims. A PROs is any report regarding patient health status referred directly by patients, without clinician or third party interpretation. Although linked, HRQoL and PRO should not be used as interchangeable terms. PROs address the source of the report, not the concept or content, and therefore represent disease effects on health and functioning from the patients’ perspective. Quite often, as we observed in this survey, patient, and doctor perspectives do not coincide (29). In an age that is rapidly moving toward “personalized medicine,” particularly in the MS field, it seems logical health-related needs as expressed by patients be incorporated to clinical trial design and outcome analysis.

We recognize the present study has limitations. First, neurologists from the SNA were invited to participate through a newsletter. Although newsletters in general have rather low online viewing rates (30), it allowed us to reach physicians of different ages, from varied regions and work places. Second, the nature of the study (email survey) only relied on subjective patient assessment, not providing meaningful information on MS severity (i.e., EDSS score). Nevertheless, several other studies have shown most aspects of MS disability are adequately self-assessed by patients (31, 32). Third, 30% of patients responding the questionnaire were unable to identify their specific clinical disease subtype. This reflects, as mentioned above, poor doctor–patient communication. And four, SF-36 has not been specifically validated for physician/patient comparison, and is thought to present certain limitations as an outcome measure in MS (33, 34). Although, other instruments specifically designed for QoL evaluation in MS exist, such as the Multiple Sclerosis International Quality of Life and Multiple Sclerosis Quality of Life-54 (MSQOL-54) questionnaires (25, 35, 36), none of them have been validated to compare doctor versus patient perception regarding the course of disease. More importantly, none of these scales have been validated in our region. This is a crucial point especially in the evaluation of cognitive function. Conversely, the SF-36 questionnaire has been validated in Argentina (18), which is why we selected it for comparison of our data to those of previous studies (10, 11).

Beyond these limitations, QoL data collected in this study are a clear example of the disagreement between MS patients and physicians in terms of assessment, and reinforce the need to enhance neurologist/patient communication. New tools like PROs are being increasingly implemented and may help us better understand real patient needs. The data reported in this article suggest moreover, that we are starting to head in the right direction.

## Ethics Statement

This study was carried out in accordance with the recommendations of “Institute Raúl Carrea, Institutional Review Board” with written informed consent from all subjects. All subjects gave written informed consent in accordance with the Declaration of Helsinki. The protocol was approved by the “Institute Raúl Carrea, Institutional Review Board.”

## Author Contributions

MY and MF contributed to the conception and design of the work. MY, MF, and MG made the acquisition of the data. MY, MF, and JC contributed to the interpretation of the data and statistical analysis. MY and JC wrote the manuscript. All authors revised the draft of the manuscript and provided important intellectual contributions.

## Conflict of Interest Statement

The authors declare that the research was conducted in the absence of any commercial or financial relationships that could be construed as a potential conflict of interest.
